# Maximizing the Wildlife Conservation Value of Road Right-of-Ways in an Agriculturally Dominated Landscape

**DOI:** 10.1371/journal.pone.0120375

**Published:** 2015-03-20

**Authors:** Robert A. McCleery, Allison R. Holdorf, Laura L. Hubbard, Brian D. Peer

**Affiliations:** 1 Department of Wildlife Ecology and Conservation, University of Florida, Gainesville, Florida, United States of America; 2 Department of Biological Sciences, Western Illinois University, Macomb, Illinois, United States of America; University of Western Ontario, CANADA

## Abstract

There has been a growing recognition that the narrow linear strips of uncultivated vegetation that lie between roads and agricultural crops, referred to as roadside right-of-ways or verges, can serve as areas for the conservation of wildlife. The features of right-of-ways that should influence the composition of wildlife communities vary considerably. Our goal was to determine what features of right-of-ways increased the conservation potential of right-of-ways for wildlife in a grassland system dominated by agricultural production. We sampled 100 right-of-ways for birds and 92 right-of-ways for small mammals in McDonough and Warren Counties in west-central Illinois. We found that the sizes of right-of-ways and the amount of traffic on the adjacent roads synergistically worked to influence wildlife communities. On roads with low traffic, avian species richness increased rapidly with increased right-of-way width, while on roads with high traffic, avian richness increased only slightly with increasing right-of-way widths. We found that wider roadside right-of-ways (preferably across the road from equally wide right-of-ways) with thicker and taller vegetation had the greatest conservation value for birds and small mammals. The features that enhanced the conservation value of right-of-ways in our study area were uncommon. Efforts to create or enhance these features for the benefit of wildlife would likely face numerous obstacles. Nonetheless, from a grassland conservation perspective, working with stakeholders to implement specific strategies to enhance these often neglected areas may be an effective complement to purchasing and restoring conservation lands away from roads.

## Introduction

The expansion and intensification of agricultural production has been the leading driver of land-use alteration across the globe [1,2]. Croplands cover at least 11% percent of the earth’s terrestrial surface, but in some ecoregions, like the temperate grasslands of the United States and the former USSR, the amount and intensity of agricultural production is much higher [3–6]. In particular, over 43% of the lands in Midwestern regions of the United States are currently covered with crops and almost all of the native prairie grasslands in the region have been replaced by row-crop agriculture [5,7]. Furthermore, most agricultural production in the developed world utilizes intense agricultural practices that eliminate unplanted vegetation, leaving entire landscapes with negligible areas of habitat for wildlife [7]. This has caused drastic declines in the populations of native wildlife in grassland systems under intensive cultivation [6,8,9].

With global landscapes increasingly dominated by intensive agriculture, there has been a growing recognition that the narrow linear strips of uncultivated vegetation that lie between roads and agricultural crops, referred to as roadside right-of-ways (ROWs) or verges, can serve as areas for the conservation of plants and wildlife [10–14]. ROWs have been shown to have conservation value for rare plants, mammals, amphibians, birds, and insects [14–17]. ROWs can increase the abundance, diversity, and nest success of birds compared with adjacent agricultural lands, as well as facilitate the movements of small mammals and carnivores [17–21]. However, ROWs vary considerably in width, vegetative structure, and disturbance regime, and it is likely that these characteristics influence the composition of wildlife communities within them [11,22]. Additionally, it is possible that the benefits of ROWs may be greatly reduced or even negated by the roads that they border. Roads are a powerful force in shaping wildlife communities and can have direct (road kill) and indirect (avoidance, contamination, noise pollution) impacts on wildlife [10,18,23–25] particularly wildlife that utilize ROWs [26]. It is likely that the vegetation structure and size of ROWs interact synergistically with road characteristics and traffic patterns to influence wildlife communities [26].

Previous research on animal diversity in ROWs has shown that birds and small mammals utilize ROWs when the vegetative structure of the ROW most closely resembles the structure of the habitats they utilize away from ROWs [11,22,27,28]. Research has also shown that the width of ROWs is related to species richness [11,28–30]. Compared with ROWs adjacent to less trafficked roads, population abundances and community diversity can decline on ROWs adjacent to roads with heavy traffic [31,32]. However, a majority of the research on animal diversity within ROWs has taken place on ROWs with woody vegetation (i.e. hedgerow, trees) or in areas that were not historically grasslands [11,27,28,30]. Findings from these studies may not be applicable to ROWs in the planet’s primary grassland systems. Furthermore, most of the research on animal conservation in ROWs has been confined to one taxonomic group, yet it is well established that using multiple taxonomic groups provides a better metric for understanding ecological processes and conservation planning [33,34].

Our goal for this study was to determine what features of ROWs increased the conservation of ROWs for two distinct wildlife (terrestrial vertebrate) communities in grasslands dominated by agricultural production. Specifically, we examined how the features of ROWs and roads influenced birds and small mammals. Small mammals play important roles in grassland systems as herbivores, seed predators, and prey for numerous predatory species [35–38]. Small mammal communities are highly dynamic and respond rapidly to changes in their environment making them useful indicators of overall ecosystem health [39]. Birds are similarly critical to the health and function of ecosystems because of their roles as seed dispersers, scavengers, and nutrient cyclers [40]. Populations of grassland birds in the northern hemisphere have precipitously declined over the past several decades [41,42]. Many species of common grassland birds have declined by >50% over the last 40 years, and some species, such as the eastern meadowlark, have decreased by 72% [43,44]. The loss of grassland birds appears to correspond with the loss of habitat at both breeding and wintering grounds, as well as with pesticide use [42,45]. Localized declines in grassland small mammals over the past two decades likewise have been attributed to land conversion and overall deterioration of the grasslands [46–48]. ROWs may provide refuge for some native ground nesting birds, birds of prey, and small mammals in grassland systems [16,19,20,22,27].

To understand the conservation potential of ROWs in grasslands in the tall-grass prairie region of the United States, we measured differences in species richness and the occurrence of indicator species among ROWs. Species richness is a standard and easily interpretable metric that is useful in identifying areas of conservation value in communities with reduced numbers of species due to anthropogenic stressors [49]. We also evaluated the occurrence of indicator species to safeguard against species richness’s potential to inflate the conservation value of areas dominated by invasive and generalist species [50–52]. Using both metrics, we predicted that wider ROWs with vegetative structure that resembled the endemic grassland of the region (tall non-woody vegetation, intermediate levels of visual obstruction, and more grass) would have greater conservation value. We predicted that ROWs adjacent to roads with more traffic would have reduced levels of richness in small mammal and bird communities. We also predicted that increased soil compaction would decrease richness of small mammals and that the presence of perches on ROW would increase avian diversity.

## Materials and Methods

### Study area

Native tall-grass prairie grasslands, characterized by their rich soils and tall (1.5–2.5 m) grasses once covered nearly two-thirds of the state of Illinois in the United States [53,54]. This fire dependent ecosystem endemic to central North America has been reduced to <1% of its former range [55]. With most of the Illinois grasslands converted for agricultural uses, some of the remaining grasslands in the state (>300,000 ha) can be found along roadsides [16,56].

**Fig 1:**
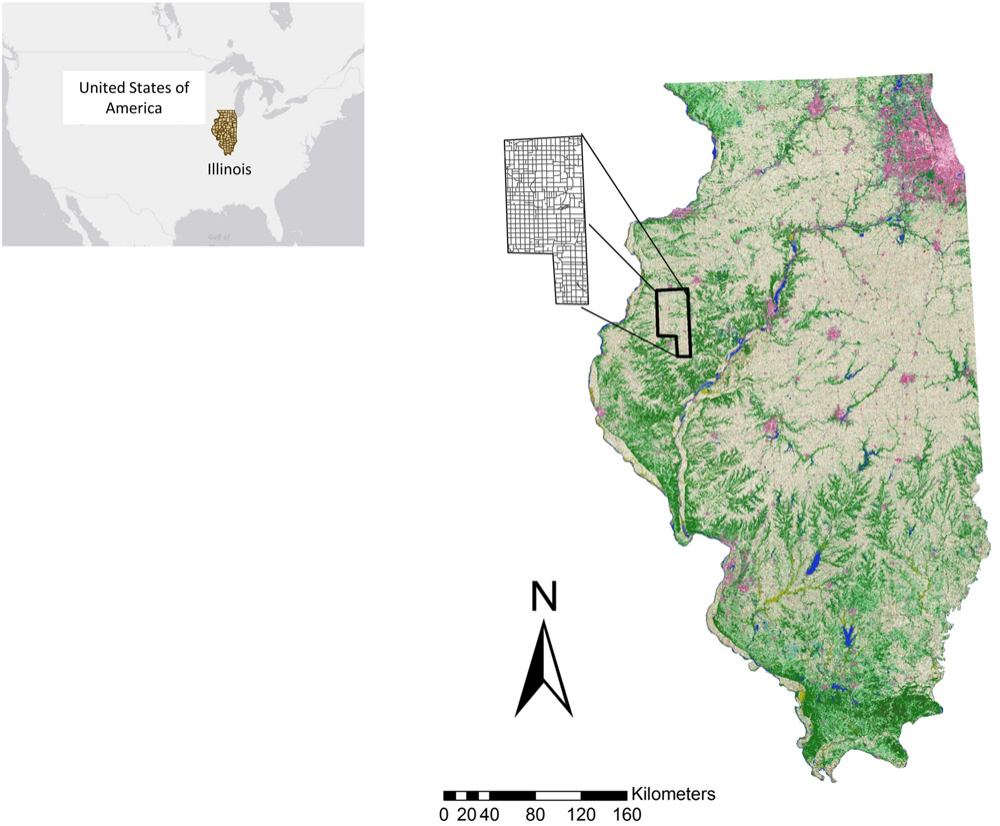
Study area. The study area included roads and roadside right-of-ways in McDonough and Warren Counties, Illinois, USA. Insert depicts the location of the State of Illinois within the USA.

Our study area covered 1329 km^2^ in portions of McDonough and Warren Counties, on the relatively flat Galesburg plain [57] in west-central Illinois (Fig. 1). Our study area was overwhelmingly rural with a population density of <18 persons per km^2^ and only one major town with a population of about 22,000 [58]. Our study area was historically dominated by tall-grass prairie but has been used for commercial agricultural production of corn (*Zea mays*) and to a lesser extent soybeans (*Glycine max*) for at least the last 40 years. These crops were usually planted the end of April and harvested mechanically in October. We worked exclusively on ROWs between agricultural crops and roads. These areas were often dominated by the non-native grasses tall fescue (*Festuca arundinacea*) and smooth brome (*Bromus inermis*). The ROWs within our study area were managed by private landowners and to a lesser extent by state and county departments of transportation. The Illinois Department of Transportation has reported mowing ROWs an average of 3 times annually [59], yet most ROWs in our study area were mowed more frequently (≥1 a month).

Common birds found in our study site included red-winged blackbirds (*Agelaius phoeniceus*), brown-headed cowbirds (*Molothrus ater*), killdeer (*Charadrius vociferus*), eastern meadowlarks (*Sturnella magna*), and dickcissels (*Spiza americana*). Prairie voles (*Microtus ochrogaster*), deer mice (*Peromyscus* spp.), and northern short-tailed shrews (*Blarina brevicauda*) were the dominant members of the small mammal communities within ROWs.

### Site selection

We randomly selected ROW sampling sites from all roads within the study area, stratified into different categories. We categorized sites into six strata based on road size and annual average daily traffic on the study site (*AADT*: data obtained from the Illinois Department of Transportation [IDOT] http://dot.state.il.us/): 1) large roads (highways/interstates with 4 lanes) with high traffic (4650–4700 vehicles per day), 2) large roads with medium traffic (100–400 vehicles per day), 3) large roads with low traffic (25–50 vehicles per day), 4) small roads (two lanes with no shoulder) with high traffic (2100–3000 vehicles per day), 5) small roads with medium traffic (100–200 vehicles per day), and 6) small roads with low traffic (0–25 vehicles per day). The distribution of traffic varied considerably between the two road sizes so we used different levels of *AADT* for large and small roads strata to capture the actual variation in traffic patterns in the study area. Each sampling site consisted of a 100m stretch of road and the bordering ROWs. To eliminate any spurious effects from features known to influence the composition wildlife communities, we only sampled sites adjacent to row-crop agriculture and >300m away from houses, forested areas, and streams.

### ROW and road characteristics

At each site, we measured features of the ROWs and of the road that we believed might influence the conservation potential of ROWs for wildlife communities. On the roads, in addition to *AADT*, we measured the width of the impervious road surface at the center of the site (*Imperv*). We took vegetative and environmental measurements within ROWs, parallel to the road at 30m, 60m and 90m and used the averages on each side of the road for our analysis. We quantified compaction (*Soil*, measured in pounds per square inch [psi], DICKEY-john Soil Compaction Tester) because small mammals, particularly fossorial and semi-fossorial ones, avoid heavily compacted soils that can discourage nests and burrows and simplify vegetation [60, 61]. We measured the average width of each ROW (*ROW*) which ranged from 3.6m to 35.3m, and collected data on the structure of the vegetation on the fore-slope and back-slope. We measured the height of vegetation in cm (*Height*) and visual obstruction (*VO*: a measure of vegetation thickness and biomass) using a Robel pole [62] marked at 0.05 m intervals and viewed from 4m away in each of the four cardinal directions. We visually estimated the herbaceous cover (*Forb*) and grass cover (*Grass*) within a circular plot (1m diameter) by placing measurements into one of six percent ranges: 1) 0%-5%, 2) 5%-25%, 3) 25%-50%, 4) 50%-75%, 5) 75%-95%, and 6) 95%-100% [63]. We also recorded the presence or absence of perches for birds (*Perch*: sign posts, fences, trees, utility poles, etc.) on ROWs.

From our measurements, we also created a number of other variables that we believed would be important in understanding the response of wildlife communities in ROWs. We generated a non-linear measure of visual obstruction by adding a quadratic term to the VO model (*VO-nonlinear* = Intercept + *VO* + *VO* ^*2*^) because grassland bird communities and populations can show non-linear responses to vegetative structure [64]. Measures of visual obstruction in a grassland system can also be used as a proxy for overall biomass [62], so we created an index of biomass for each ROW by multiplying the average visual obstruction by the width of the ROW (*Biomass*: VO*ROW). To address the considerable variation in structure and the size of ROWs on opposing sides of the roads and it is possible that the combined size of both ROWs (*ROWtotal*) influenced wildlife communities that readily cross roads. Finally, we created a variable that gave equal weight to increasing ROW width and decreasing AADT to evaluate the potential of synergistic effects between these features (SYN = *ROW* Standardized from 1–0 [highest to lowest] * *ADDT* Standardized from 0–1 [highest to lowest]. For example, this variable allowed us to determine if wildlife communities could only thrive in large ROWs with reduced traffic rates.

### Small mammals

We sampled small mammal communities on 92 ROWs from 46 sites (7–9 sites per strata) during the growing season from 26 May-18 August 2010. We sampled small mammals on each side of the road with transects of 10 Sherman live traps placed 10m apart (SFAL Folding Trap, H.B. Sherman Traps, Inc., Tallahassee, Florida, USA). Additionally, we placed pitfall trapping arrays at the end of each transect to help with the detection of shrews (*Soricidae*). We used silt fencing to create a drift fence in a V formation on opposing sides of a 5 gallon bucket [65]. We baited the live traps with bird seed (black oil sunflower seeds, cracked corn, millet, and milo) and peanut butter and trapped for three nights, checking the traps each morning. We recorded the species, sex, weight, tail length, body length, and foot length of each capture and marked them with a unique identifier (ear-tagged [1005–1, National Band Co., Newport, Kentucky, USA] or with a distinct nail polish pattern for shrews) and released them where they were captured. We did not differentiate between *Peromyscus maniculatus* and *Peromyscus leucopus* because of difficulty distinguishing between the two without molecular techniques [66]. All of our procedures were approved by the Western Illinois University Institutional Animal Care and Use Committee (IACUC, 10–51) and the Illinois Department of Natural Resources (scientific research permit NH10–5313), and performed in accordance with the American Society of Mammalogy guidelines [67] to ensure the ethical treatment of animals. Permission to access ROWs was granted by McDonough and Warren counties, Illinois; however, neither county required or had the ability to issue a permit to aces these areas.

### Birds

We sampled bird communities on 100 ROWs from 50 sites, sampling 8–9 sites from each strata based on road size and traffic patterns. We sampled each ROW twice with two independent observers for a total of four independent transect surveys. We started each survey with two observers positioned on the edge of the same ROW and clearly visible to one another (3.6m to 35.3m apart). The observers synchronized stopwatches and after a 10 minute acclimation period they walked the same 100m transect in unison and then repeated this procedure on the adjacent ROW. During each 10-minute transect survey, each observer independently recorded the species and time of each bird they saw. We recorded the total number of bird detections by both observers and used the timing of each detection to calculate the number of unique bird sightings and the amount of agreement among observer detections. We conducted surveys from 20 minutes before sunrise to 3 hours after sunrise during the breeding season from 10 May-18 August 2010. To avoid any spurious effects of weather we did not survey if mist or rain was present, or if wind speeds exceeded 17km/hr. Upon completion of each transect survey, each observer dragged a rope through their transect to flush and record any ground nesting birds that were missed during the initial survey [68].

## Statistical Analyses

### Species richness

We compared the total number of species detected (species richness) on each ROW from equal sampling efforts [69] and evaluated factors that influenced species richness on ROWs using generalized linear mixed models fitted to Poisson distributions. First, we examined the relationships among variables and removed a variables when correlation coefficient’s > 0.70. Then we developed a suite of *a priori* candidate models to predict the influence of ROWs on the richness of birds (19 models) and small mammals (17 models) that included the additive effects of vegetation height (*Height*), visual obstruction (*VO*), non-linear response to visual obstruction (*VO-nonlinear*), ROW width (*ROW*), the combined width of ROWs on both sides of the road (*ROWtotal*), soil compaction (*Soil*), forb cover (*Forb*), grass cover (*Grass*), vegetative biomass (*Biomass*), average annual daily traffic (*AADT*), the presence or absence of perches (*Perch*) and the synergistic effects of ROW width and average annual daily traffic (*SYN*), as well as intercept only and global models. Each model included site as a random variable to account for the lack of independence between the two ROWs at each site (one on each side of the road).

We evaluated all models using the lme4 package [70] in R 2.14.1 (R Development Core Team 2011), which ranks models for their relative quality according to the Akaike Information Criterion corrected for small sample size (AICc), where the preferred model is the one with the minimum AICc value. We ranked the models based on the change in AICc values relative to the lowest-scoring model (ΔAICc) [71]. Models ≤4 AICc units were considered competitive models. Models that were >4 AICc units were rejected as likely predictor models. After selecting the top models, we averaged the beta (*β*) estimates of variables in the models and determined the variables’ predictive importance by inspecting its 95% confidence intervals to make sure they did not contain zero.

### Indicator species

In addition to examining species richness, we used occurrence of indicator species as a metric of the conservation value of ROWs to the grassland ecosystem. We selected three species associated with grasslands in the tall-grass prairie ecoregion: one mammal, the western harvest mouse (*Reithrodontomys megalotis*), and two birds, the dickcissel and eastern meadowlark. Western harvest mice are predominately seed eaters associated with grasslands and are found in areas associated with taller non-woody vegetation [72]. Similarly, the dickcissel, an omnivore, and eastern meadowlark, an insectivore, are obligate grassland specialists that prefer areas with considerable grass cover [73,74].

Each of these three species was relatively uncommon on the ROWs (<30%) and not observed in the adjacent agricultural field, so we examined the factors that influenced their probabilities of occurrence within the ROWs. We converted capture nights for small mammals (n = 3) and independent observer surveys for birds (n = 4) into encounter histories for each ROW. We evaluated factors that influenced the probability of occurrence using an occupancy modeling approach [75] that accounted for the probability of detection (*p*) when estimating their probability of occurrence (*ψ*). We specifically used the Bayesian occupancy modeling approach outlined by Kéry [76] because it allowed us to fit complex models to our hierarchical data and accounted for the lack of spatial independence between data collected on opposite sides of the same road [76–78]. Accordingly, we fit each model with a random variable on the occupancy component of the model account for site effects [78]. First, we evaluated model parameters that might affect *p*. For the harvest mouse we looked at categorical parameters that accounted for different detection rates for each trap night (*trap night*) and just the first trap night (*first night*). For birds, we evaluated a single parameter that accounted for differences in detection between the 2 morning surveys (*survey*). Then using the best model for *p* we evaluated models of occurrence that included the following biological important variables described above: *ROW*, *Perch*, *VO*, *AADT*, *ROWtotal*, *Grass*, *Forb*, *Biomass*, *Soil*, and *Imperv*. We standardized continuous covariates before analysis to improve model convergence [76]. We used non-informative priors (mean = 0, variance = 0.001) for all coefficients surveyed and ROW covariates. We fit our models using WinBUGS 1.4 [79] but coded the models using the R package R2WinBUGS [80]. We initiated Markov Chain Monte Carlo (MCMC) runs for each model with the following settings: iterations = 20,000; burn-in period = 5000 iterations; number of chains = 3; and thinning rate = 3. We assessed the convergence of the MCMC chains using by visual assessment of trace plots in WinBUGS and examined the Rhat statistic to insure it was near one. We selected models using the stepwise approach, checking whether the 95% credible intervals (CRIs) covered zero to determine if the parameter was warranted in the model [74,81]. We used this approach because the Bayesian deviance information criterion (DIC) that is most often used as the model selection criterion [82] has been shown to perform poorly with hierarchical models [83,84].

## Results

### Small mammals

**Fig 2:**
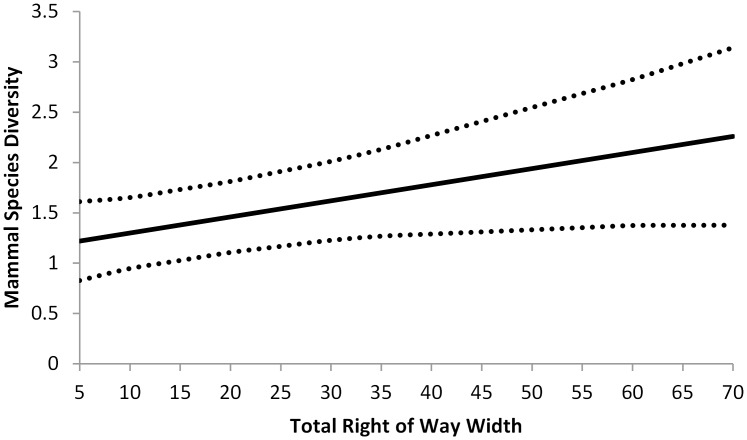
Predictive values and 95% CI of species richness of small mammals on roadside right-of-ways in west-central Illinois. Values are a function of the combined width of roadside right-of-ways on both sides of the road (*ROWtotal*).

From a total of 2,760 trap nights, we captured 312 individuals of nine different species the great majority of which were native (> 94%; Table 1). The best competing model to explain small mammal richness had one variable, the combined width of the trapped and adjacent ROWs (*ROWtotal*; Table 2). As *ROWtotal* increased, so did the diversity of the small mammal community (*β* = 0.0117, 95% CI = 0.0002–0.0233), with species richness increasing by a biologically significant 1 species only after 60m (Fig. 2). Competing models (< 4 AICc units from the best model) ranked above the null model (Table 2) included measures of visual obstruction (*VO*), vegetation height (*Height*) and ROW width (*ROW*). Visual obstruction decreased richness (*β* = -0.0087, 95% CI = -0.0495–0.0322) and *Height* (*β* = 0.2778, 95% CI = -0.1334–0.6888) and *ROW* (*β* = 0.0196, 95% CI = -0.0086–0.0477) increased richness, but all three variables had 95% CI that included zero, suggesting that they were not strong predictors of mammal diversity on ROWs. Furthermore, all of the variables in competing models (< 4 AICc units from the best model) ranked below the null model also had 95% CI that included zero providing little support for our predictions that *Grass*, *AADT*, or soil compaction (*Soil*) would influence small mammal richness. We removed *Imprev* from the analysis because it was highly correlated (R = 0.77) with ROW width.

**Table 1:** Total number of small mammal individuals captured on 92 roadside right-of-ways from 46 sites in west-central Illinois, USA, 26 May-18 August 2010.

Species	Number of Captures	Number of Individuals
Deer mice (*Peromyscus* spp.)	65	52
House mouse (*Mus musculus*)	12	11
Prairie vole (*Microtus ochrogaster*)	58	47
Meadow vole (*Microtus pennsylvanicus*)	24	22
Northern short-tailed shrew (*Blarina brevicauda*)	164	156
Cinereus shrew (*Sorex cinereus*)	1	1
Western harvest mouse (*Reithrodontomys megalotis*)	8	8
Thirteen-lined ground squirrel (*Spermophilus tridecemlineatus*)	12	10
Brown rat (*Rattus norvegicus*)	5	5
Total	349	312

**Table 2:** Candidate models examining factors influencing the species richness of small mammals on roadside right-of-ways (ROWs) in west-central Illinois, USA.

Canidate Models	k	AICc	ΔAICc	AICcWt
*ROWtotal*	3	288.9304	0	0.1668
*VO*+*H*	4	289.5764	0.646	0.1208
ROW	2	289.8383	0.9079	0.1060
*Intercept*	3	290.3023	1.3719	0.0840
ROW*AADT	3	290.9025	1.9721	0.0622
*VO*	3	291.1971	2.2667	0.0537
*Grass*	3	291.237	2.3066	0.0526
*Forb*	3	291.2809	2.3506	0.0515
*VO-nonlinear*	4	291.2935	2.3631	0.0512
*ROW*+*AADT*	4	291.3835	2.4531	0.0489
ROW*AADT+*ROW*	4	291.8913	2.961	0.0380
*AADT*	3	291.9269	2.9965	0.0373
*Soil*	3	292.0062	3.0758	0.0358
*Biomass*	3	292.1691	3.2387	0.0330
*Height*	3	292.4371	3.5067	0.0289
*Perch*	3	292.4373	3.507	0.0289
Global	9	296.6329	7.7025	0.0003

Number of parameters (k), AICc, change in AICc (ΔAICc), and AICc weights (AICcWt) are reported. Models include variables for the height of non-woody vegetation (*Height*), visual obstruction (*VO*), non-linear response to visual obstruction (*VO-nonlinear*), ROW width (*ROW*), the combined width of ROWs on both sides of the road (*ROWtotal*), soil compaction (*Soil*), forb cover (*Forb*), grass cover (*Grass*), an index of vegetative biomass (*Biomass*), average annual daily traffic (*AADT*), synergistic effect of ROW width and traffic (*SYN*), and the presence or absence of perches for birds (*Perch*).

**Fig 3:**
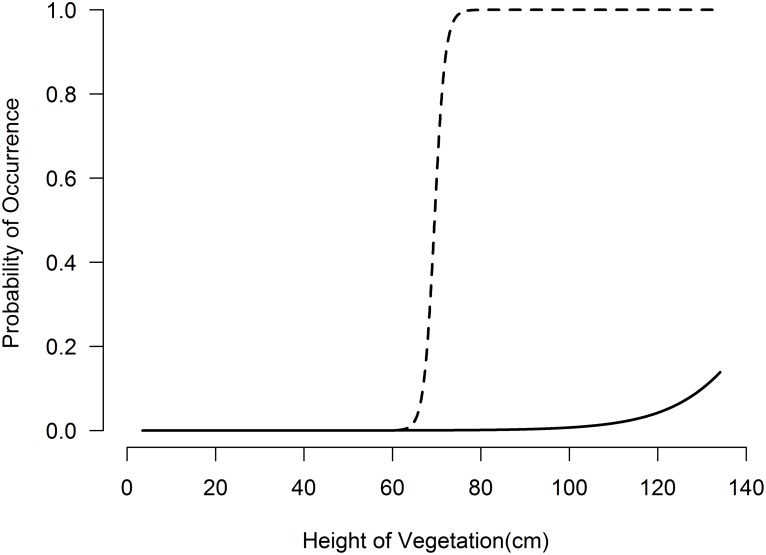
Predictive values of harvest mouse (dashed line) and meadowlark (solid line) occurrence on roadside right-of-ways in west-central Illinois as a function of vegetation height (*Height*).

**Fig 4:**
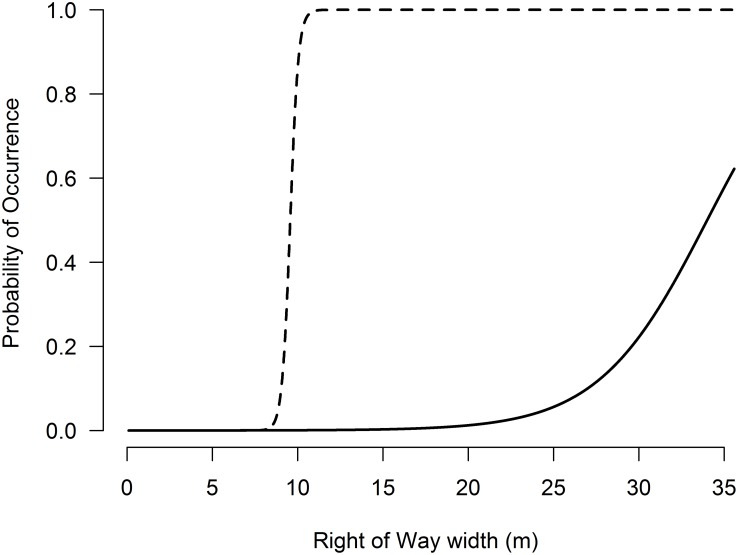
Predictive values of harvest mouse (dashed line) and dickcissel (solid line) occurrence on roadside right-of-ways in west-central Illinois as a function of right-of-way width on one side of the road (*ROW*).

Our models for the occurrence of harvest mouse included vegetation height (*Height*) as an explanatory variable (posterior mean = 28.62, CRI = 1.23–73.92), with ψ rising rapidly with non-woody vegetation over 75cm (Fig. 3). The best model also included the width of the ROW as a variable, with harvest mouse occurrence increasing with increased ROW width (posterior mean = 34.77, CRI = 5.98–86.23). The probability of harvest mouse occurrence increased to >50% after 9m ROW width and approached 100% after 11m ROW width (Fig. 4). Our best model of occurrence for harvest mice within ROWs had a posterior mean occupancy (ψ) of 0.37 (95% CRI = 0.26–0.56) and posterior mean detection (*p*) of 0.08 (95% CRI = 0.03–0.15) per night of trapping, indicating overall probabilities of occurrence and detection on the study area of 37% and 8%, respectively.

### Birds

**Fig 5:**
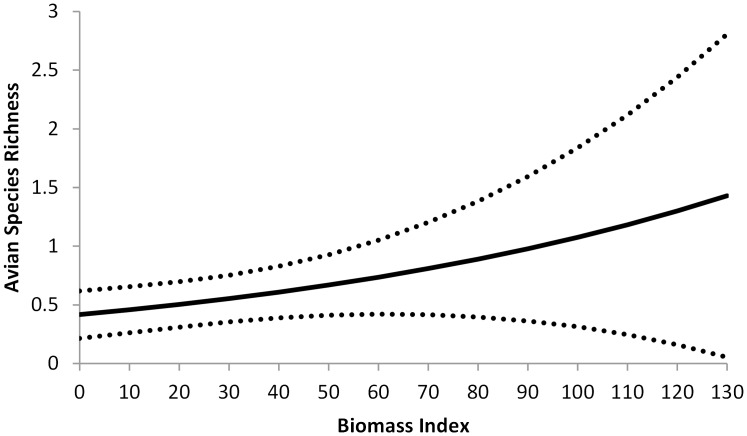
Predictive values and 95% CI of avian species richness on roadside right-of-ways in west-central Illinois as a function of an index of vegetative biomass available on the right-of-way (*Biomass*).

We recorded 15 native birds species and no introduced invasive species for a total of 831 detections and 424 sightings (birds recorded at a unique time in the survey), with 97.5% agreement among observers (Table 3). All birds jointly detected were identified as the same species. The best competing model to explain avian species richness included the synergistic effects of ROW width and traffic (*SYN*) and the availability of perch sites (*Perch*, Table 4). Models (ΔAICc < 4) competing with the best model also included additive effects of *ROW*, *ADDT*, *ROWtotal* and the overall vegetative biomass (*Biomass*) on the site (Table 4). Averaging parameter estimate across all models the—*SYN* (*β =* 2.7170, 95% CI = 0.0734–5.361), *Biomass* (*β* = 0.0094, 95% CI = 0.00002–0.0189), and *Perch* (*β* = 0.5221, 95% CI = 0.0103–1.0340)—showed positive relationships between model variables and avian richness. ROWs with places for birds to perch (*Perch*, *S* = 0.77, 95% CI = 0.48–1.06) had a slight increase in richness compared to areas without a perch (*S* = 0.44, 95% CI = 0.24–0.64). Vegetative biomass also had an influence on avian richness, with species richness ranging from 0.4 to 1.4 across the range of biomass values (Fig. 5). ROW width (*β =* 0.0302, 95% CI = -0.02003–0.0984) the combined width of ROWs on both sides of the road had (*β =* 0.0226, 95% CI = -0.0034–0.0486), and AADT (*β* = -0.2467, 95% CI = -0.6464–0.1531) all had 95% CI that included they were not strong predictors of avian richness.

**Table 3:** Total number of bird detections and sightings (bird recorded at a unique time in survey) from surveys of 100 roadside right-of-ways from 50 sites in west-central Illinois, USA, 10 May-18 August 2010.

Species	Detections	Sightings
American goldfinch (*Spinus tristis*)	6	3
Barn swallow (*Hirundo rustica*)	14	7
Indigo bunting (*Passerina cyanea*)	4	2
Chipping sparrow (*Spizella passerina*)	4	2
Brown-headed cowbird (*Molothrus ater*)	133	68
Dickcissel (*Spiza americana*)	39	20
Eastern meadowlark (*Sturnella magna*)	41	21
Common grackle (*Quiscalus quiscula*)	8	4
American kestrel (*Falco sparverius*)	4	2
Killdeer (*Charadrius vociferous*)	85	44
Mourning dove (*Zenaida macroura*)	8	4
Northern rough-winged swallow (*Stelgidopteryx serripennis*)	28	14
Red-winged blackbird (*Agelaius phoeniceus*)	445	228
Upland sandpiper (*Bartramia longicauda*)	4	2
Western kingbird (*Tyrannus verticalis*)	8	4
Total	831	425

**Table 4:** Candidate models examining factors influencing the species richness of birds on roadside right-of-ways in west-central Illinois, USA.

Models	k	AICc	Delta_AICc	AICcWt
*SYN*+*perch*	4	247.4687	0	0.3041
*ROW*+*Perch*	4	249.3127	1.844	0.121
*ROW*+*Perch*+*AADT*	5	249.6286	2.1598	0.1033
*SYN*	3	249.6542	2.1854	0.102
*Biomass*	3	250.6263	3.1575	0.0627
*ROW*	3	251.1275	3.6588	0.0488
*ROWtotal*	3	251.2503	3.7815	0.0459
*SYN*+*ROW*	4	251.4130	3.9443	0.0423
*ROW*+*AADT*	4	251.9359	4.4671	0.0326
*Perch*	3	252.0613	4.5925	0.0306
Intercept	2	252.2513	4.7826	0.0278
*Height*	3	253.4778	6.0091	0.0151
*Grass*	3	253.6568	6.1881	0.0138
*Forb*	3	253.8968	6.4281	0.0122
*Soil*	3	254.0766	6.6079	0.0112
*AADT*	3	254.3554	6.8867	0.0097
*VO-nonlinear*	4	254.6785	7.2097	0.0083
*VO*+*Height*	4	255.0656	7.5969	0.0068
Global	10	257.7743	10.3055	0.0018

Number of parameters (k), AICc, change in AICc (ΔAICc), and AICc weights (AICcWt) are reported. Models include variables for the height of non-woody vegetation (*Height*), visual obstruction (*VO*), non-linear response to visual obstruction (*VO-nonlinear*), ROW width (*ROW*), the combined width of ROWs on both sides of the road (*ROWtotal*), soil compaction (*Soil*), forb cover (*Forb*), grass cover (*Grass*), an index of vegetative biomass (*Biomass*), average annual daily traffic (*AADT*), synergistic effect of ROW width and traffic (*SYN*), and the presence or absence of perches for birds (*Perch*).

**Fig 6:**
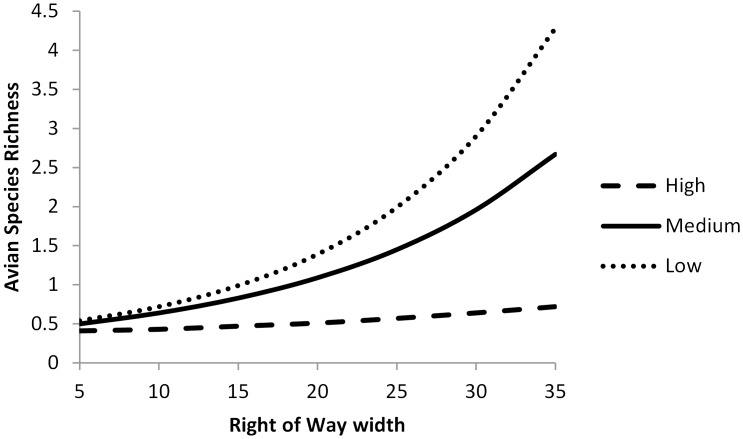
Predictive plot of avian species richness on roadside right-of-ways in west-central Illinois as a function of the interactive effect of right-of-way width (*ROW*) and average annual daily traffic (*AADT*). Avian species richness is plotted versus the value of *ROW* predicted at three *AADT* strata: low (1, dotted line), medium (1427, solid line) and high (7000, dashed line).

We did find relatively strong support for the benefits of ROW width and the negative influence of traffic on avian richness. When we held other variables constant, we found that bird species richness increased substantially (by ≥1 species) on ROWs >20m wide (Fig. 6). To understand the relationship between ROW width and traffic, we plotted the predicted values of avian richness as a function of ROW width at low (1), medium (1427) and high (7000) strata of *AADT*. We found the influence of ROW width on richness decreased with higher rates of traffic (Fig. 6). On roads with low traffic, avian richness increased from <1 on ROWs narrower than 10m to >4 on ROWs wider than 30m. On roads with high traffic, avian richness only increased from <0.5 on ROWs narrower than 10m to <0.75 on ROWs wider than 30m. Additionally, we found that richness decreased with increased traffic (*ADDT*, *β* = -0.0001, 95% CI = -0.0003–0.0001), but the 95% CI of this variable included zero, suggesting that it alone was not a strong predictor of avian richness.

Like the harvest mouse, meadowlark occurrence was linked to the structure of vegetation within the ROW. The best model for meadowlark occurrence included one variable, height of non-woody vegetation *(Height*, posterior mean = 2.75, CRI = 0.601–7.212), with meadowlark occurrence increasing once the height of non-woody vegetation was >100cm (Fig. 3). The best model for explaining the occurrence of dickcissels only included width of the ROW (*ROW)*, with the probability of dickcissel occurrence increasing considerably on ROWs greater than 24m wide (Fig. 3).

From our best models, we found the estimates of dickcissel and meadowlark occurrence on ROWs in the study area were similar and relatively low at 7% (meadowlarks ψ = 0.07 [95% CRI = 0.07–0.10]; dickcissels ψ = 0.07 [95% CRI = 0.07–0.09]). Nonetheless, our ability to detect these birds was quite high, near 70% (meadowlarks p = 0.68 [95% CRI = 0.43–0.86]; dickcissels p = 0.70 [95% CRI = 0.50–0.86]). The best model for meadowlarks included a variable accounting for differences in detection between the first and second visits to the ROWs (posterior mean = 0.401, CRI = 0.015–2.187), whereas the model for dickcissels did not.

## Discussion

Our research suggests that ROWs within agriculturally dominated temperate grasslands can provide habitat for native wildlife. The conservation value of ROWs appears to be a function of the ROW’s size, vegetative structure, and traffic patterns on adjacent roads. Previous research has shown that increased traffic can limit bird communities on ROWs [32,85] and that most across taxonomic groups respond positively to increased ROW width (small mammals [27]; butterflies [86]; birds [16]), but research has not clarified how these opposing patterns interact. We found that the widths of ROWs and the amount of traffic on the adjacent road worked synergistically to influence avian communities, but not small mammals. On roads with low traffic, avian species richness increased rapidly with increased ROW width, while on roads with high traffic, avian richness increased only slightly with increased ROW width.

The mechanism that limited richness of birds but not small mammals on ROWs adjacent to more heavily trafficked roads was unclear. The reduced richness of birds was possibly due to their sensitivity to the noise and artificial light on heavily trafficked roads [85]. Artificial light may reduce habitat quality of ROWs [87], but lighted roads were not common on our rural study area. Noise travels farther from roads in open grassland environments [88] and can hinder bird communication, making the ROW an undesirable habitat [85]. More directly, birds are susceptible to vehicle collisions on heavily trafficked roads [85]. If collisions were the reason for reduced avian richness, large ROWs with heavy traffic could create ecological traps [89] where birds perceive the larger ROWs as suitable habitat but suffer reduced levels of fitness due to vehicular mortalities. Like a handful of other studies, we found no evidence that increased traffic altered small mammal richness [22,90]. Our inability to link changes in small mammal richness to traffic patterns may have been a function of the relatively light traffic on our study area. However, studies with higher rates of traffic have similarly found no impact on small mammal communities [90,91]. Therefore, it is more likely that traffic simply did not have a measureable influence on small mammal richness. The different responses of birds and small mammals to traffic patterns clearly illustrates the difficulty in generalizing the results from a single taxonomic group to all wildlife within ROWs and highlights the need for a comprehensive understanding of ROWs that accounts for the mechanisms influencing wildlife conservation within them.

A pattern that was consistent across taxonomic groups was a favorable response to increased width of ROWs. Wider ROWs increased bird and small mammal richness as well as the occurrence of indicator species. It has been well established in conservation biology that the conservation potential of patches of remnant vegetation increases with increased size [92–94]. Larger patches have larger core areas and are less likely to be influenced by forces of the external environment (edge effects) and more likely to have heterogeneity in vegetative structure that attracts more diversity wildlife communities [93]. Furthermore, larger patches of remnant vegetation with larger populations are more likely to retain species and are less likely to have localized extinction events [93,94]. However, finding landscapes with ROWs wide enough to have a meaningful influence on wildlife conservation may prove difficult in regions dominated by intensive agriculture. On the random sample of ROWs in our study area, the majority (62%) of ROWs were less than 7m wide and only 23% of ROWs greater than 10m wide. According to our models, wider ROWs would only lead to a biologically meaningful increase in bird and small mammal richness (>1 additional species) on a small portion of our sites (6% and 4% respectively).

Contrary to our predictions that the ROWs with grass cover and vegetative structure (height and thicknesses) similar to the endemic tall-grass prairies of the region would have increased species richness, we found only a relatively small influence of vegetative structure on the richness (biomass) of bird and small mammal communities. One possible explanation for this result is that while the structure of ROWs may be similar to the endemic prairies, the species composition of most ROWs in our study area was dominated by invasive grasses. Planting of native prairie grasses on ROWs has been shown to increase the diversity of birds [95], bees [14] and butterflies [13]; it is possible that the absence of these species limited the conservation value of the ROWs on our study area. Even without native grasses, the structure of vegetation on our ROWs did have a marked influence on indicator species. Two of the three indicator species responded to increased height of vegetation, with harvest mice increasing when vegetation was >75cm and eastern meadowlarks increasing with vegetation >100cm (Fig. 3). These findings are consistent with the habitat preferences of these two species [71,73] and suggest that the vegetative structure of ROWs is likely important for grassland endemic wildlife.

## Conclusions

Temperate grasslands around the world have been greatly reduced and transformed for the production of crops and other agricultural uses [1,2,5]. As a result of this land conversion, native wildlife populations and communities have rapidly declined [7–9]. By providing habitat for endemic wildlife, ROWs have the potential to aid in the conservation of grassland wildlife in landscapes dominated by intensive agriculture. ROWs may increase species richness and the occurrence of endemic indicator species, yet the conditions conducive to conservation may be limited within the agricultural environment. We found that wider ROWs (preferably across the road from equally wide ROWs) with thicker and taller vegetation and adjacent to roads with low traffic volume provided the best available conditions for small mammal and bird communities adjacent to agricultural crops. Nonetheless, to determine if the observed increases in species richness and indicator species occurrence under these condition were ultimately beneficial or detrimental (i.e. ecological traps) to wildlife populations we recommend intensive demographic studies.

The easiest way to potentially enhance the height and thickness of vegetative structure of ROWs is to reduce mowing during the growing season. Reducing the frequency of mowing on ROWs has been suggested as a way to increase the diversity of butterflies, moths, and bird nesting success [13,16,82,96], and may have the added benefit of reducing management costs on ROWs [97,98]. Currently, wide ROWs are more commonly found on heavily trafficked roads, thus another way to improve the wildlife conservation value of ROWs would be to create wider ROWs in areas with low traffic volume. The creation of these types of ROWs would reverse the typical pattern of placing wider ROWs adjacent to larger roads with higher traffic volumes. Wide ROWs next to roads with high traffic volume do not increase species richness and hold the potential to create ecological traps and harm bird communities. We suggest that some of the resources placed into acquiring and managing ROWs on heavily trafficked roads may be more useful if redirected towards ROWs adjacent to less trafficked roads.

We realize that ROWs are functionally important in draining water and that roadside safety will always be the first priority when considering management practices and design of these areas; however, it may still be possible to implement a practice that favors increased wildlife diversity, like reducing mowing on ROWs. Altering mowing regimes may be difficult to implement because government agencies are often hesitant to adopt policies that contradict cultural norms and public perceptions. Research suggests that the public often favors highly manicured ROWs to unmowed ROWs [97]. These preferences are likely shaped by a lack of information about the costs, aesthetics, and environmental value of ROW management practices like mowing [97]. Thus, it may be possible to utilize outreach and education strategies to change stakeholders’ perceptions and to eventually change management practices on ROWs [97]. Any such efforts in our study area would need to be focused on the individual farm operators that manage many ROWs on secondary roads. With cropland prices in the Midwestern United States at over $2,800 per ha ($7000 per acre) and rising [99], working with the public to enhance these relatively marginal areas may provide a cost-effective complement to purchasing and restoring grasslands away from roads.

## Supporting Information

S1 Dataset Bird Richness. Bird species richness and environmental variables by site.
(XLSX)

S2 Dataset Mammal Richness. Mammal species richness and environmental variables by site.
(XLSX)

S3 Dataset Bird Occupancy. Meadowlark and Dicksissel occupancy by site.
(XLSX)

S4 Dataset Mammal Occupancy. Harvest mouse occupancy by site.
(XLSX)
